# Camalexin-Induced Apoptosis in Prostate Cancer Cells Involves Alterations of Expression and Activity of Lysosomal Protease Cathepsin D

**DOI:** 10.3390/molecules19043988

**Published:** 2014-04-02

**Authors:** Basil Smith, Diandra Randle, Roman Mezencev, LeeShawn Thomas, Cimona Hinton, Valerie Marah

**Affiliations:** 1 Center for Cancer Research and Therapeutic development, Department of Biological Sciences, Clark Atlanta University, Atlanta, GA 30314, USA; 2 Department of Biology, Georgia Institute of Technology, Atlanta, GA 30332, USA; 3 Department of Biological Sciences, Florida A & M University, Tallahassee, FL 32307, USA

**Keywords:** camalexin, prostate cancer, lysosomes, cathepsin D, phytoalexins

## Abstract

Camalexin, the phytoalexin produced in the model plant *Arabidopsis thaliana*, possesses antiproliferative and cancer chemopreventive effects. We have demonstrated that the cytostatic/cytotoxic effects of camalexin on several prostate cancer (PCa) cells are due to oxidative stress. Lysosomes are vulnerable organelles to Reactive Oxygen Species (ROS)-induced injuries, with the potential to initiate and or facilitate apoptosis subsequent to release of proteases such as cathepsin D (CD) into the cytosol. We therefore hypothesized that camalexin reduces cell viability in PCa cells via alterations in expression and activity of CD. Cell viability was evaluated by MTS cell proliferation assay in LNCaP and ARCaP Epithelial (E) cells, and their respective aggressive sublines C4-2 and ARCaP Mesenchymal (M) cells, whereby the more aggressive PCa cells (C4-2 and ARCaPM) displayed greater sensitivity to camalexin treatments than the lesser aggressive cells (LNCaP and ARCaPE). Immunocytochemical analysis revealed CD relocalization from the lysosome to the cytosol subsequent to camalexin treatments, which was associated with increased protein expression of mature CD; p53, a transcriptional activator of CD; BAX, a downstream effector of CD, and cleaved PARP, a hallmark for apoptosis. Therefore, camalexin reduces cell viability via CD and may present as a novel therapeutic agent for treatment of metastatic prostate cancer cells.

## 1. Introduction

**Figure 1:**
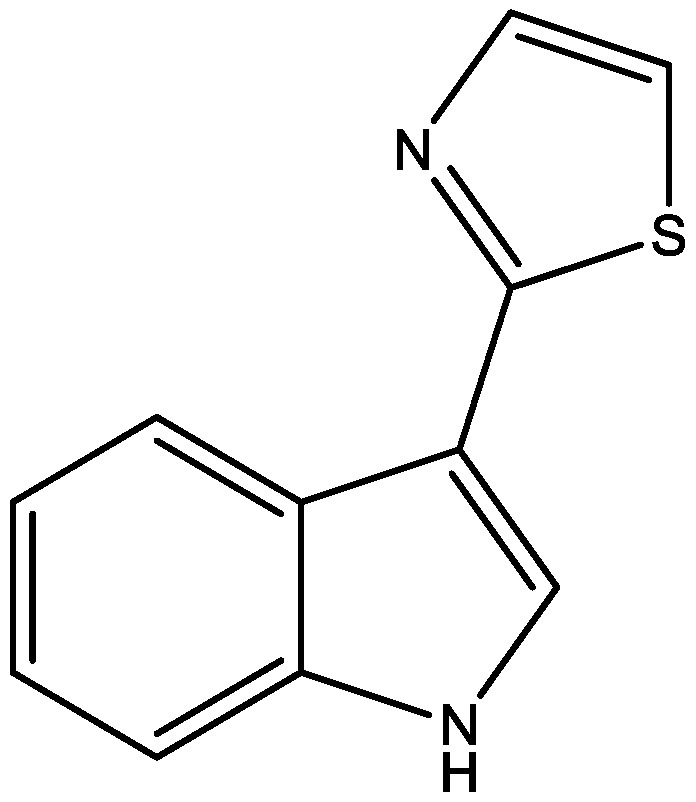
The structural formula of camalexin.

Among cancer-related deaths in men, prostate cancer is the second-leading cause in the United States despite recently observed decrease in mortality [1]. Once cancers metastasize to other organs, a majority of patients die from their tumors as opposed to other causes [2], and this is due in part to the tumor ultimately becoming androgen-independent within a median of 18 to 24 months after castration [3]. Current treatments have proved inadequate in controlling prostate cancer, and search for novel therapeutic agents for the management of this disease has become a priority for researchers. The discoveries of the chemopreventive and chemotherapeutic properties of phytochemicals have generated considerable interest among cancer researchers. Camalexin (Figure 1) is an indole phytoalexin produced in various cruciferous plants upon exposure to environmental stress and plant pathogens [4,5,6,7]. 

It has been shown to possess moderate antifungal and bacteriostatic, as well as antiproliferative and cancer chemopreventive properties [7,8]. Our laboratory has shown that camalexin induced apoptosis in prostate cancer cells (PCa) through the generation of Reactive Oxygen Species (ROS), and that the more aggressive prostate cancer cells with higher levels of endogenous ROS displayed greater sensitivity to camalexin treatments evidenced by decreased viability and increased apoptosis as compared to the less aggressive prostate cancer cells, while normal epithelial cells were unaffected [9]. The generation of intracellular ROS activates several signal transduction pathways leading to inflammation, cell cycle progression, apoptosis, migration, and invasion in cancer [10]. Thus, excessive production of ROS or inadequacy in a cell’s antioxidant defense system (or both) induces oxidative stress with consequent initiation of cellular processes associated with initiation and development of many cancers including prostate cancer [11]. Hydrogen peroxide has been shown to increase progressively in the lesser aggressive LNCaP prostate cancer cells to its more aggressive sublines C4, C4-2 and C4-2B, with concomitant increase in tumorigenic and metastatic potential [12]. Hence, several studies were conducted on cancer treatments focusing on ROS inhibition with limited success [13]. Conversely, in many preclinical models of cancer, excessive ROS generation triggers pro-apoptotic pathways with subsequent apoptosis [14]. Thus, the therapeutic use of pro-oxidants to target prostate cancer cells is gaining traction in cancer research [15]. Therefore, compounds that are capable of inducing ROS especially in cancer cells warrant further investigation.

Apoptosis induced by oxidative stress has been observed in several studies and includes activation of cell cycle genes such as p53 and p21 [16,17,18]. Key players in the regulation of apoptotic cell death are the mitochondria, where they coordinate caspase activation through release of cytochrome C. Lysosomes, the major cell digestive organelles containing numerous hydrolases that degrade intracellular and extracellular materials delivered, has been implicated in the regulation of cell death [19]. Common to both mitochondria and lysosomes is their increased membrane permeability resulting in release of their contents in the early phase of apoptosis [20]. Varying stimuli induces lysosomal membrane permeabilization (LMP) with translocation of enzymes from lysosomal compartment to cytosol, such as oxidative stress [21,22], TNF-α [23] and p53 [24]. Among the hydrolytic enzymes released, the cathepsins were found to participate in apoptosis following their release into the cytosol. In oxidative stress-induced apoptosis, release of cathepsin D (CD) from lysosomes and induction of apoptosis could be prevented by the antioxidant α-tocopherol [21]. Deiss *et al.* have shown that CD anti-sense RNA protected Hela cells from IFN-γ- and Fas-induced cell death [25]. Additionally, it has been demonstrated that p53 accumulates rapidly after oxidative stress and has two binding sites located at the CD promoter gene, and that CD participates in p53-dependent apoptosis [26]. The role of CD in apoptosis has been linked to lysosomal release of mature CD into the cytosol, in turn leading to mitochondrial release of cytochrome c into the cytosol [27,28,29,30], activation of pro-caspases-9 and -3 [31,32], *in vitro* cleavage of Bid [33], or Bax activation independent of Bid cleavage [34]. Pepstatin A, an aspartate protease inhibitor of CD, could partially delay apoptosis induced by oxidative stress [28,29,30,31], or even when it was co-microinjected with CD [32]. Therefore, CD could play a key role in apoptosis mediated by its catalytic activity.

Previously, we have shown that prostate cancer cells expressing high levels of ROS (C4-2 and ARCaPE cells stably overexpressing Snail) displayed further increase in ROS upon camalexin treatment which led to decreased viability and increased apoptosis through activation of caspase-3 and -7 [9]. Interestingly, the less aggressive cells (LNCaP and ARCaPE with empty vector) were less responsive to camalexin and could be induced to be more responsive by addition of exogenous hydrogen peroxide [9] thus showing that camalexin mediates its response via ROS. In this current study, we have dissected the mechanism of camalexin-induced (apoptosis) decreased-cell viability further and shown for the first time that it is mediated through CD. Hence, in our experiments, we utilized two prostate cancer progression models, LNCaP/C4-2 and ARCaPE/ARCaPM and found that camalexin reduced cell viability in PCa cells that involved relocation of CD from lysosomes to cytosol, and increased protein expression of p53, mature CD, Bax, and cleaved PARP. Moreover, pepstatin A, the peptide inhibitor of CD activity was able to reverse the effects of camalexin. Targeting lysosomal proteases such as CD may therefore provide a great therapeutic potential in especially metastatic prostate cancer.

## 2. Results and Discussion

### 2.1. Camalexin Treatments Decreases Cell Proliferation in the More Aggressive Prostate Cancer Cells as Compared to the Lesser Aggressive Cells

**Figure 2:**
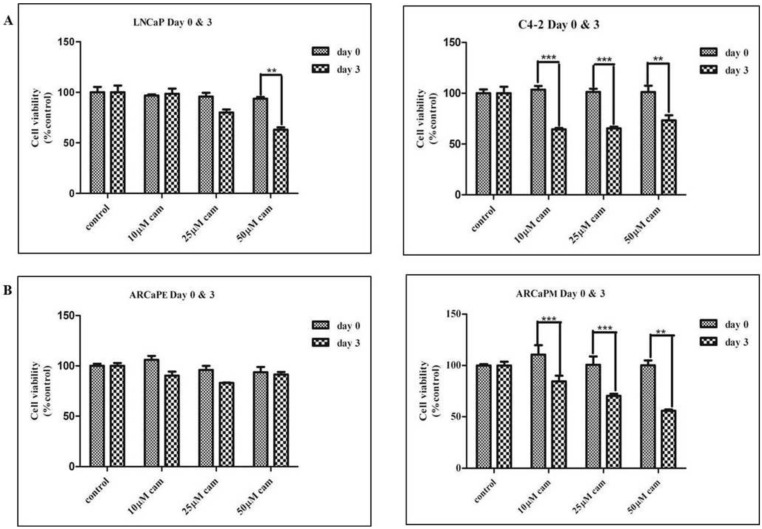
The more aggressive C4-2 and ARCaPM prostate cancer cells are more sensitive to camalexin as compared to LNCaP and ARCaPE cells. Viability was determined using MTS proliferation assay at day 0 or day 3 for LNCaP and C4-2 (**A**), and ARCaPE and ARCaPM (**B**) cells treated with 10, 25 and 50 μM camalexin. Statistical analysis was done using ANOVA and Tukey’s Multiple Comparison as Post Hoc (* p < 0.05, ** p < 0.01, *** p < 0.001). Values were normalized to untreated controls and expressed as mean ± S.E.M (N = 3).

Previously, we have shown that camalexin was more potent in reducing cell viability in C4-2 as compared to LNCaP cells suggesting that camalexin was more potent in the more aggressive cell line [9]. Confirming these results utilizing CellTiter 96^®^ AQueous One Solution Cell Proliferation Assay (MTS assay) we observed that at day 0 for both LNCaP and C4-2 cells, viability was unaffected but on day 3, only 50 µM camalexin decreased cell viability in LNCaP by approximately 40% ± 2% (*p* < 0.01), while camalexin decreased C4-2 cell viability by approximately 40% ± 2% (*p* < 0.001) for 10 and 25 μM camalexin and 30% ± 5% (*p* < 0.01) for 50 μM camalexin, respectively (Figure 2A). We also tested the effect of camalexin on ARCaPE (epithelial) and ARCaPM (mesenchymal) cell lines that are derived from the same parental ARCaP but represent an EMT progression model [35,36]. CellTiter 96^®^ AQueous One Solution Cell Proliferation Assay (MTS assay) was utilized and the results for day 0 and day 3 represented. For day 0, both ARCaPE and ARCaPM cells showed no change in viability as expected, however, on day 3, camalexin treatment of ARCaPE decreased cell viability by approximately 23% (*p* < 0.05) at 25 µM treatment only, while for ARCaPM cells 10, 25 and 50 µM decreased viability by approximately 22% ± 5% (*p* < 0.05), 28% ± 1% (*p* < 0.01) and 47% ± 1% (*p* < 0.001), respectively (Figure 2B). Therefore, we show that the more aggressive C4-2 and ARCaPM cells displayed greater sensitivity to camalexin treatment than the lesser aggressive LNCaP and ARCaPE cells.

### 2.2. Camalexin Treatment of Prostate Cancer Cells Increases Protein Expression of the Lysosomal Protease CD, Bax and p53 Transcription Factor

**Figure 3:**
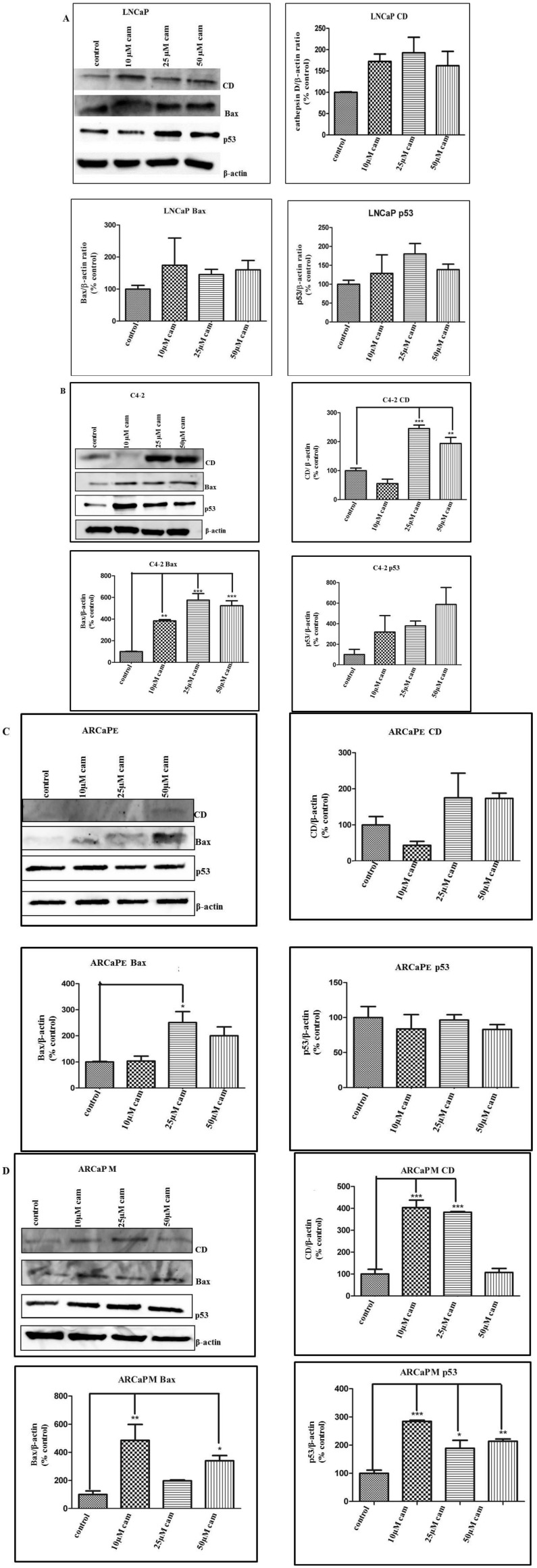
Camalexin leads to increased expression of CD, Bax, and p53 protein in prostate cancer cell lines. Western Blot analysis was performed to examine CD, Bax and p53 protein expression following exposure of LNCaP (**A**) and C4-2 (**B**), ARCaPE (**C**), ARCaPM (**D**) cells to various doses of camalexin for 3 days. β-actin was used as loading control. Densitometry was performed for each Western Blot using Image J Software (National Institutes of Health). Data are representative of at least 3 independent experiments and statistical analysis was done using ANOVA and Tukey’s Multiple Comparison as Post Hoc (* p < 0.05, ** p < 0.01, *** p < 0.001).

Previously, our laboratory has shown that camalexin treatment of prostate cancer cells produces oxidative stress-induced apoptosis with consequent increased caspase 3 activity and PARP cleavage [9]. Survey of the literature revealed that several agents and molecules of endogenous origin can induce lysosomal membrane permeabilization, among which ROS are the most important [37,38]. As a result, CD is translocated from the lysosomes to the cytosol and triggers a rapid change in Bax conformation together with insertion of this protein to the outer mitochondrial membrane [34]. CD and BAX protein expression was analyzed by western blot analysis in camalexin-treated (10, 25 and 50 µM) ARCaPE, ARCaPM, LNCaP and C4-2 cells. Camalexin treatments of LNCaP cells showed a tendancy towards increased protein expression of CD at the 10, 25 and 50 µM camalexin treatments, whilst Bax showed no significance in protein expression levels of treated *versus* untreated control cells (Figure 3A). However, for C4-2 cells, camalexin treatments significantly increased protein expression of CD (25 and 50 µM treatments, *** *p* < 0.001, ** *p* < 0.01 respectively) and BAX protein (10, 25 and 50 µM treatments, ** *p* < 0.01, *** *p* < 0.001, *** *p* < 0.001 respectively) as compared to the untreated control cells (Figure 3B). In ARCaPE cells, no significant levels of CD protein expression were noted for untreated control, 10 and 25 µM camalexin treatments, while at 50 µM treatment there was a tendency toward increased expression although there was no statistical significance noted (Figure 3C). At 25 µM camalexin treatment only, significant increase in Bax protein expression was noted (* *p* < 0.05) in ARCaPE cells (Figure 3C), although at 50 µM camalexin treatment there is a strong tendency towards increased expression as compared to untreated control cells (Figure 3C). In ARCaPM cells, however, 10 and 25 µM camalexin treatments induced significant increased protein expression of CD (*** *p* < 0.001) and BAX (10 and 50 µM treatments, ** *p* < 0.01, * *p* < 0.05 respectively) *vs.* the untreated control cells (Figure 3D). At 25 µM camalexin treatment of ARCaPM cells, the data showed increased Bax protein expression level when compared to the untreated control cells (Figure 3D). Wu *et al.*, has demonstrated that p53 accumulates in cells subsequent to oxidative stress and can bind the CD promoter gene, and thus CD can participate in p53-dependent apoptosis [26]. Because we saw increased CD protein expression levels subsequent to camalexin treatments in our cells we decided to further investigate whether its trans-gene p53 may also be involved in the induced stress response. Western blot analysis showed that LNCaP and C4-2 cells at 25 and 50 µM camalexin treatments produced increased p53 protein expression when compared with the untreated control cells (Figure 3A,B). ARCaPE cell lysates did not display significant alterations in expression of p53 protein subsequent to camalexin treatments (Figure 3C). However, ARCaPM cell lysates showed significant increase in p53 protein expression at 10, 25 and 50 µM camalexin-treatments (*** *p* < 0.001, * *p* < 0.05, and ** *p* < 0.01) respectively (Figure 3D). Overall, our data shows that camalexin increases p53, CD and BAX expression, which are proteins involved in apoptosis and the effect is more pronounced in the ARCaPM and C4-2 cells as compared to ARCaPE and LNCaP cells, respectively.

### 2.3. Camalexin Shifts Subcellular Localization of CD from the Lysosome to the Cytosol

**Figure 4:**
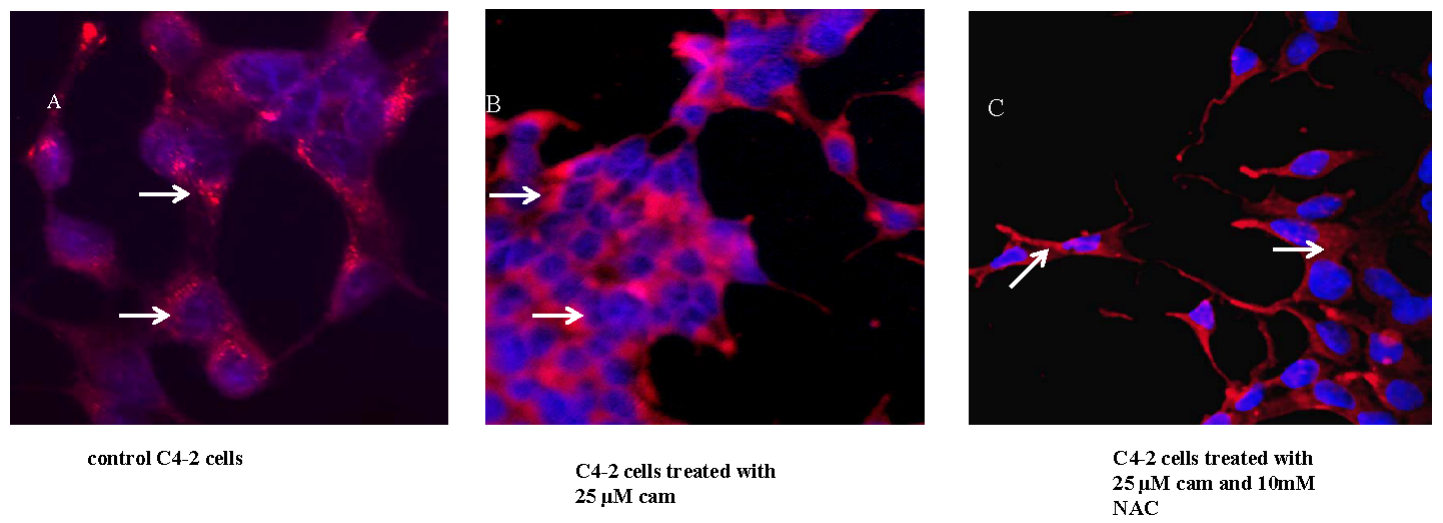
Camalexin exposure in C4-2 cells induces release of lysosomal enzymes to the cytosol. CD localization was analyzed by Immunofluorescence analysis in C4-2 untreated (**A**), treated with 25 μM camalexin (**B**) or 25 μM camalexin and 10 mM NAC for 24 h (**C**). CD in untreated C4-2 cells appears punctuated in intact lysosomes while camalexin treatment led to more diffuse staining depicting CD release into cytosol. Co-treatment with NAC increased punctuated staining similar to untreated control cells.

A significant amount of hydrogen peroxide is produced in the mitochondria subsequent to oxidative stress and is able to diffuse into the lysosomes where ferruginous material delivered by autophagy is accumulated, resulting in Fenton-type reaction and production of highly reactive hydroxyl radicals [39]. These radicals cause lipid peroxidation of lysosomal membrane and subsequent leakage of proteases into the cytosol. In this way oxidative stress from the mitochondria is amplified with the help of redox-active iron-rich lysosomes [40,41]. The role of CD in apoptosis has been defined previously [25,26] and using immunocytochemistry, we analyzed CD localization within cells following camalexin treatments. We detected granular staining of CD in C4-2 untreated control cells depicting its location within intact lysosomes (Figure 4A). After 24 h camalexin treatment only, fluorescence staining for CD was more diffuse, indicating its translocation from the lysosomes to the cytosol of C4-2 cells (Figure 4B). Co-treatment of camalexin with the ROS scavenger, N-acetylcysteine (NAC), produced increased granular staining almost similar to the untreated cells suggesting that it could reverse in part, the effects of camalexin (Figure 4C). Therefore, camalexin not only increases CD expression but also leads to its leakage from the lysosome to the cytoplasm through increased ROS production.

### 2.4. NAC Abrogates Camalexin-Mediated Increased p53, Bax and CD Protein Expression in Prostate Cancer Cells

**Figure 5:**
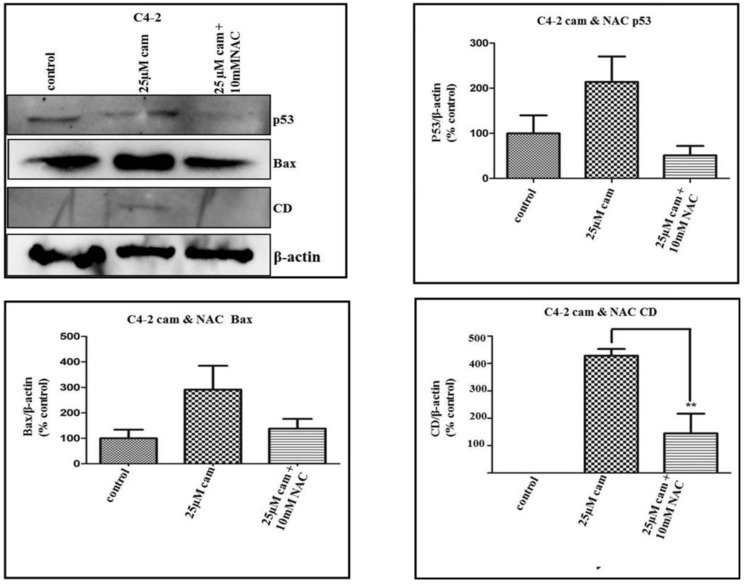
NAC abrogates the effects of camalexin-mediated p53, Bax and CD protein expression. C4-2 cells were treated with 25 μM camalexin or 25 μM camalexin plus 10 mM NAC for 3 days. Western blot analysis was done to examine p53, Bax and mature CD protein expression. Treatment for 3 days indicated that NAC could inhibit p53, Bax and CD protein expression when combined with 25 μM camalexin. β-Actin was used as loading control and densitometry of protein expression assessed for treated *versus* untreated control cells. Data are representative of at least 3 independent experiments. Statistical analysis was done using ANOVA and Tukey’s Multiple Comparison as *Post Ho*c (** p < 0.01).

Oxidative stress has been shown to be responsible for lysosomal membrane destabilization with consequent permeabilization and leakage of proteases into the cytosol [40]. The ROS scavenger NAC was added to camalexin-treated C4-2 cells and p53, Bax and CD protein expression assessed *via* western blot analysis. The results strongly indicated decreased expression of p53 and Bax, and significant abrogation of CD protein expression (** *p* < 0.01) by co-treatment of cells with NAC (Figure 5). Hence, camalexin utilizes oxidative stress to induce p53, Bax and CD protein expression.

### 2.5. Pepstatin A, a CD Inhibitor, Antagonizes Camalexin-Mediated Decrease in Cell Viability, and Its Promotion of Pro-Apoptotic Protein Expression

**Figure 6:**
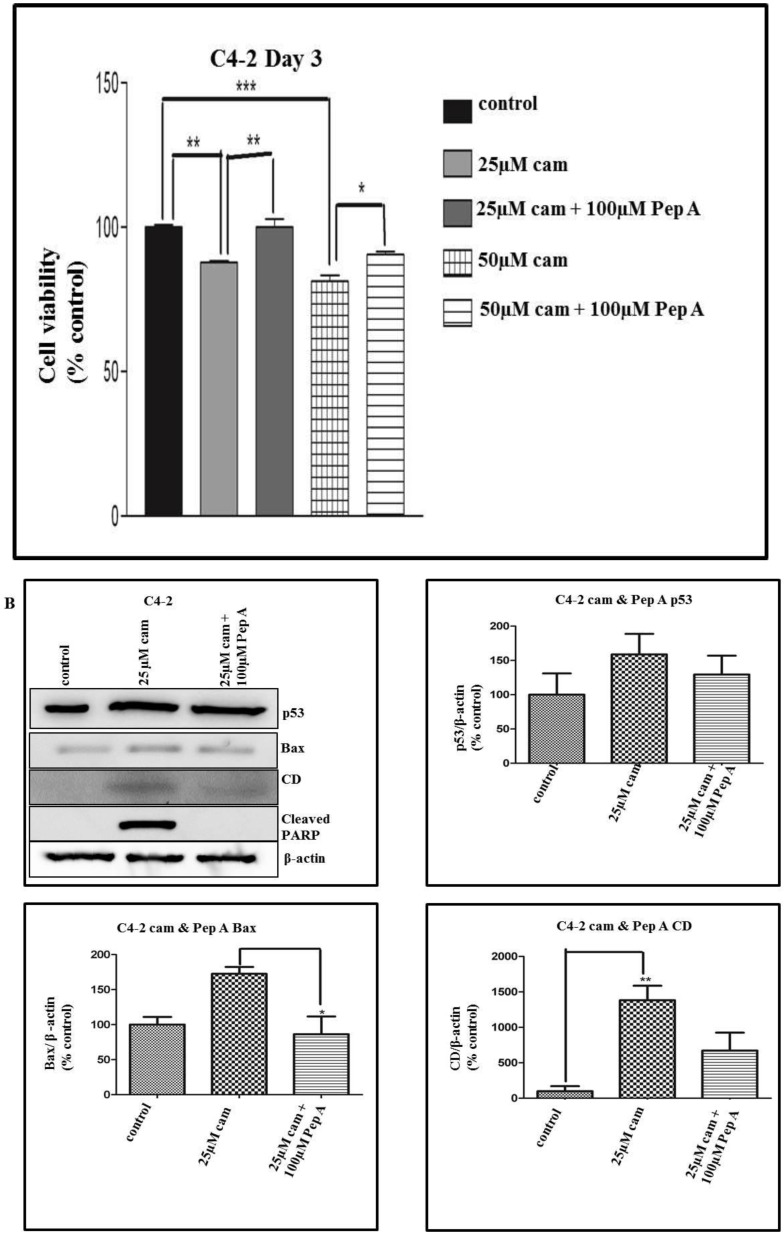
Pepstatin A abrogates the effects of camalexin-mediated decrease in cell viability and PARP cleavage. C4-2 cells were treated with 25 and 50 μM camalexin only, or 25 and 50 μM camalexin plus 100 μM Pep A for 3 days and cell viability assayed using the MTS proliferation assay **(A)**. Western blot analysis was done to examine p53, CD and cleaved PARP protein expression in untreated, camalexin-treated and camalexin plus Pep A- treated C4-2 cells, along with the densitometry of protein expression (**B**). Treatment for 3 days indicated that Pep A could significantly inhibit camalexin-mediated decrease in cell viability and increase in Bax and cleaved PARP protein expression, but does not significantly alter p53 and CD protein expression. Statistical analysis was done using ANOVA and Tukey’s Multiple Comparison as a *Post Hoc* Test. Values were expressed as mean ± S.E.M normalized to untreated controls, and 25 μM camalexin *vs.* 25 μM camalexin + 100 μM Pep A (* p < 0.05, ** *p* < 0.01). (N = 3). β-Actin was used as loading control and data are representative of at least three independent experiments.

We utilized the potent peptide inhibitor of CD activity, pepstatin A (Pep A), to confirm the role of this protease in camalexin-induced apoptosis in prostate cancer cells. We observed after 3 days that camalexin-mediated decrease in cell viability was blocked significantly by co-treatment with 100 µM Pep A plus 25 or 50 μM camalexin in C4-2 cells (Figure 6A). Additionally, treatment of C4-2 cells with Pep A significantly antagonized camalexin-mediated increase in Bax and PARP cleavage protein expression without significantly affecting p53 levels (Figure 6B). Hence, Pep A functions downstream of CD and our studies imply that p53 must be acting upstream of CD since it is not significantly affected by Pep A. 

### 2.6. Discussion

The lysosomal pathway of apoptosis involves partial lysosomal membrane permeabilization (LMP) with subsequent release of cathepsins into the cytosol [19,42]. Resistance of cancer cells to chemotherapy is often considered to involve decreased sensitivity to apoptosis, and therefore alternate pathways to induce cell death in resistant tumors are highly demanded [43]. Hence, lysosomal cell death represents an alternate pathway where released cathepsins can activate apoptosis or execute apoptosis-like cell death independent of the caspases [44,45,46]. We have previously shown that camalexin decreases cell viability in prostate cancer cells by induction of oxidative stress leading to apoptosis [9]. Similarly, Mezencev *et al.* has shown that camalexin-induced apoptosis in Jurkat T leukemia cells occurred via increased ROS and involve mitochondrial superoxide generation evidenced by increased MitoSOX™ staining [47]. In this report we show that camalexin may also utilize the lysosomal pathway involving CD. We showed that camalexin decreased cell viability in ARCaPE and ARCaPM prostate cancer cell lines in a concentration-dependent manner and was more potent in ARCaPM cells. This would make camalexin a potential therapeutic agent for aggressive prostate cancer and we have previously shown that it does not affect normal prostate epithelial cells [9]. Camalexin-induced cell death in ARCaPE, ARCaPM, LNCaP and C4-2 prostate cancer cells is accompanied by Lysosomal Membrane Permeability as evidenced by increased CD protein expression in treated *vs.* untreated control cells. Additionally, immunocytochemical analysis of camalexin-treated C4-2 cells displayed diffuse staining for CD *vs.* punctuated staining in lysosomes for untreated control, indicating this protease release into the cytosol. The addition of NAC to the camalexin-treated cells, displayed increased punctated staining verifying increased CD retention within lysosomes and hence lysosomal membrane stabilization. CD has been discovered as a key mediator of apoptosis induced by several apoptotic agents including oxidative stress [27,28,29,30,31,48]. Its role in apoptosis has been linked to lysosomal release of its mature form (33 kDa) into the cytosol, activation of Bax [34], and in turn leading to mitochondrial release of cytochrome c into the cytosol [27,28,29,30].

Our data reveals increased Bax protein expression in camalexin-treated prostate cancer cell lines. Mounting evidence points to Bax and other pro-apoptotic family members as central regulators of the release of proteins from the mitochondrial intermembrane space. Bax overexpression in cells or the addition of purified recombinant Bax directly to isolated mitochondria triggers release of cytochrome c into the cytosol [49,50] with subsequent activation of procaspases 9 and 3 [28]. Immuno-electron microscopy has confirmed that Bax can directly insert into the lysosomal membrane during staurosporine treatment of fibroblasts [20]. Additionally, CD has two binding sites at its promoter region for the p53 transcription factor and can participate in p53-dependent apoptosis [27]. Moreover, p53 can localize to the mitochondrial membrane and can trigger mitochondrial membrane permeabilization (MMP) by direct interaction with Bcl-2 proteins [51,52,53,54], and also can promote Bax-mediated MMP through transcriptional upregulation of BH3-only-domain proteins Puma and Noxa [55]. Hence in our model of camalexin-induced apoptosis in prostate cancer cells via oxidative stress-induction involving lysosomal protease CD, increased p53 protein expression may function as an amplification loop in the death pathway.

In order to further demonstrate that camalexin is exerting its effects on CD through ROS, co- treatment of C4-2 cells with the antioxidant NAC decreased CD translocation to the cytosol and protein expression. We further attempted to highlight the role of CD in camalexin-induced apoptosis by pretreatment of cancer cells with its known peptide inhibitor pepstatin A (Pep A). Camalexin-mediated decrease in cell viability, increase in Bax protein levels and PARP cleavage was antagonized by cotreatment of cells with Pep A. Again, studies have shown that Pep A partially delayed the apoptosis induced by oxidative stress [30] or when Pep A was co-microinjected with CD in fibroblasts [32]. These authors have therefore evidenced CD’s key role in apoptosis via its catalytic activity. 

## 3. Experimental

### 3.1.Reagents and Antibodies

Growth media RPMI 1640 (1 × with l-glutamine, and without l-glutamine and phenol red), and penicillin-streptomycin were from Mediatech Inc. (Manassas, VA, USA). Fetal bovine serum (FBS) and charcoal/dextran treated FBS (DCC-FBS) were from Hyclone (South Logan, UT, USA). T-media was from Gibco by Life Technologies Corporation (Grand Island, NY, USA). Trypsin /EDTA was from Mediatech, Inc. MTS Cell Titer 96^®^ Aqueous One Solution reagent was from Promega Corporation (Madison, WI, USA). Camalexin (40 mM in absolute ethanol) was kindly provided by our collaborator Roman Mezencev of Georgia Institute of Technology (Atlanta, GA, USA) and was synthesized as described previously [8]. N-acetylcysteine (NAC) and mouse monoclonal anti-human actin antibody were from Sigma-Aldrich Inc. (St. Louis, MO, USA). Rabbit monoclonal anti-cleaved PARP and Bax primary antibody were from Cell Signaling Technology, Inc. (Danvers, MA, USA). Goat polyclonal anti-cathepsin D and p53 primary antibody, and, donkey anti-goat HRP and were from Santa Cruz Biotechnology (Santa Cruz, CA, USA). HRP conjugated sheep anti-mouse, donkey anti-rabbit secondary antibodies, and the Enhanced Chemiluminescence (ECL) western blotting detection reagent were from GE Healthcare UK Ltd. (Buckinghamshire, UK). Nitrocellulose membranes were from Bio-Rad Life Sciences Research (Hercules, CA, USA). 

### 3.2. Cell Lines and Culture

Human prostate cancer cell line ARCaPE, an epithelial cell line and ARCaPM the more highly metastatic and mesenchymal cell line were all derived from parental ARCaP cells [35] and utilized in these experiments (a kind gift from Dr Leland Chung, Cedars-Sinai Medical Center, Los Angeles, CA, USA). They were cultured in T-media supplemented with 10% (*v/v*) fetal bovine serum (FBS), 2 mM l-glutamine, 50 µg/mL penicillin and 100 µg/mL streptomycin. Additionally, we utilized LNCaP cells from American Type Culture Collection (Manassas, VA, USA) with lesser metastatic potential and its more aggressive subline C4-2 cells (a kind gift from Dr Leland Chung). LNCaP and C4-2 cells were routinely cultured in RPMI 1640 medium supplemented with 10% (*v/v*) fetal bovine serum (FBS), 2 mM l-glutamine, 50 µg/mL penicillin and 100 µg/mL streptomycin. They were grown to 70% confluence in 95% air, 5% CO_2_ humidified incubator at 37 °C, and routinely passaged using 0.05% Trypsin/EDTA solution. For all experimental conditions RPMI 1640 without l-glutamine and phenol red and supplemented with 5% dextran charcoal stripped FBS (DCC-FBS) was used. 

### 3.3. Cell Viability Assay

ARCaPE, ARCaPM, LNCaP and C4-2 cells were plated at a density of 2,000 cells per well in 96-well plates and allowed to attach overnight. Camalexin (10, 25 and 50 µM) were added to cells and allowed to incubate at 37 °C in a humidified 5% CO_2_ atmosphere for 0 through 5 days and then viability assessed daily using the CellTiter 96^®^ Aqueous One Solution Cell Proliferation Assay according to supplier’s protocol. For cathepsin D activity inhibition studies, C4-2 cells were treated with 25 or 50 µM camalexin alone or in combination with 100 µM pepstatin A for 0 and 3 days and then cell viability assessed. 

### 3.4. Western Blot Analysis

Western blot was performed as described previously [36]. Briefly, cells were plated at a density of 2 × 10^6^ cells in 75 cm^2^ flasks, and allowed to attach overnight. Prior to treatments, ARCaPE and ARCaPM cells were serum-starved in serum-free and phenol red free RPMI medium overnight, while LNCaP and C4-2 cells were serum-starved for 4 h. Cells were then lysed with buffer containing 1 × RIPA, with protease inhibitors (aprotinin 0.1 mg/mL, PMSF 1 mM, leupeptin 0.1 mM, pepstatin A 0.1 mM) and phosphatase- inhibitor (10 mM sodium orthovanadate) and centrifuged at 13,000 rpm. Total protein content was determined using Pierce^®^ BCA Protein Assay Kit (Thermo Scientific, Rockford, IL, USA) following protocol as per manufacturer’s specifications. Cell lysates (40–50 µg) were subjected to SDS-Polyacrylamide gel electrophoresis (SDS-PAGE) and then subsequently transferred to pure nitrocellulose membrane. Western blot analysis for CD, Bax, cleaved-PARP and p53 protein expression was then performed. Anti-mouse, anti-goat and anti-rabbit IgG horseradish peroxidase in blocking solution (5% nonfat milk in TBS-Tween 20) was employed as secondary antibodies for chemiluminescent detection. Blots were visualized with chemiluminescence ECL detection system (Pierce, Rockford, IL, USA) and analyzed using FUJIFILM LAS 3000 imager and dark room x-ray film to observe protein expression. To evaluate protein loading, membranes were immediately stripped with Restore ™ Western Blot Stripping Buffer (Thermo Scientific) and reprobed for β-actin as a loading control. Densitometry of protein expression relative to untreated control cells was evaluated using ImageJ Software (National Institutes of Health, Bethesda, MD, USA). 

### 3.5. Immunocytochemistry (ICC)

When C4-2 cells reached 60%–80% confluence, 2 × 10^3^ cells were plated into 16 well chamber slides (Bio-Tek, Nunc, Winooski, VT, USA). Cells were serum-starved for 4 h, and then left untreated, treated with 25 or 50 µM camalexin, or 25 or 50 µM camalexin plus 10 mM NAC for 24 h. The media was removed and 200 µL of equal parts per volume of methanol/ethanol was added for 5 min at room temp for fixation. Methanol/ethanol was gently removed and the slides were washed 3 × 5 min with 1 × PBS and 50 µL of protein blocking solution (Dako, Camarillo, CA, USA) was added per well for 10 min at room temp. Blocking solution was drained off with a tissue and 50 µL of goat anti-CD primary antibody (1:50 or 1:100) (in Dako antibody diluent solution,) was added per well for 1 h at room temp. Slides were washed 3 × 5 min with commercial 1 × TBS-T (Dako), and then incubated with Texas red anti-goat secondary antibody in the dark for 1 h at room temp. Slides were briefly dipped into double deionized water, incubated with DAPI (1 µg/mL) for 5 min at room temp in the dark. Slides were washed 3 × 5 min with 1 × TBS-T mounted using Fluorogel mounting medium (Electron Microscopy Sciences, Hatfield, PA, USA). Slides were placed in a container covered with foil and left to dry overnight in the dark. Fluorescence microscopy was performed using AxioZeiss (Rel 4.8) fluorescence microscopy and images were captured at 20x magnification. 

### 3.6. Statistical Analysis

Statistical analysis was performed using ANOVA and Tukey’s Multiple Comparison Test as *Post-Hoc* test from Graph Pad Prizm3 software (GraphPad Software Inc., San Diego, CA, USA); * *p* < 0.05, ** *p* < 0.01, *** *p* < 0.001 were considered significant. Multiple experiments were performed in replicates of 3. 

## 4. Conclusions

Considerable progress has been made in understanding the role of lysosomes and lysosomal proteases in the apoptotic pathways [56]; however, the exact mechanisms of lysosomal membrane permeabilization and its consequences are still not well understood. Although there are supporting data for camalexin ability to generate ROS in cells [9,47], the mechanism(s) involved are yet to be elucidated. We have attempted here to show the role of mature CD in camalexin-induced apoptosis via oxidative stress-induction in prostate cancer cells. Therefore, targeting lysosomes can provide a great therapeutic strategy in cancer cells because not only do they trigger apoptosis, but also can suppress autophagic flux which starves these cells and thus can sensitize them to chemotherapeutic agents [56]. Hence, camalexin treatment of especially metastatic prostate cancer cells in combination with current chemotherapeutic agents may provide a very vital therapeutic option.
